# Proteomic analysis of plasma extracellular vesicles reveals mitochondrial stress upon HTLV-1 infection

**DOI:** 10.1038/s41598-018-23505-0

**Published:** 2018-03-26

**Authors:** Patricia Jeannin, Thibault Chaze, Quentin Giai Gianetto, Mariette Matondo, Olivier Gout, Antoine Gessain, Philippe V. Afonso

**Affiliations:** 1 Unité d’Epidémiologie et Physiopathologie des Virus Oncogènes, Département de Virologie, Institut Pasteur, Paris, F-75015 France; 2 Centre National de la Recherche Scientifique (CNRS) UMR 3569, Paris, F-75015 France; 3 Proteomics platform, Mass Spectrometry for Biology, Institut Pasteur; CNRS USR 2000, Paris, F-75015 France; 4 Bioinformatics and Biostatistics Hub, C3BI, Institut Pasteur; CNRS USR 3756, Paris, F-75015 France; 5 Service de Neurologie, Fondation Ophtalmologique Adolphe de Rothschild, Paris, F-75019 France

## Abstract

Extracellular vesicles (EVs) can participate in intercellular communication and pathogenesis. EVs contain many cargos, including proteins, and the composition of EVs differs between cell-types and activation levels. Thus, plasma EVs can be used as a biomarker of systemic response to infection and/or disease progression. In this study, we aimed at describing alterations in the protein content of plasma EVs upon infection with the human T-lymphotropic retrovirus type 1 (HTLV-1). HTLV-1 is the etiological agent of a lymphoproliferative disease (ATL) and a series of inflammatory diseases, including a neurodegenerative inflammatory disease (HAM/TSP). We found that plasma EVs are more abundant and smaller in HTLV-1 asymptomatic carriers or HAM/TSP patients when compared to uninfected healthy donors. Moreover, EVs from HTLV-1 infected donors contain markers of metabolic and mitochondrial stress.

## Introduction

Cells release vesicles that can participate in intercellular communication^1^. Indeed, extracellular vesicles (EVs) contain biologically active material, which may alter the biology of recipient cells. EVs have been shown to modulate immune response, inflammation, and viral-induced pathogenesis^2,3^. The composition of EVs differs between cell-types and activation levels; plasma EVs have been used as biomarker of infection or disease^4^. Besides these functions, EVs can also be a cellular means to remove misfolded or aggregated proteins, and non-functional organelles^5^.

The human T-lymphotropic virus type 1 (HTLV-1) is estimated to infect 5–20 million people worldwide^6^. Among HTLV-1 infected individuals, 90–95% remain asymptomatic throughout their lives. Nevertheless, HTLV-1 is the etiological agent of many severe diseases, ranging from an aggressive lymphoproliferation, the adult T-cell leukemia/lymphoma (ATL), to inflammatory syndromes, such as a neurodegenerative disease called HTLV-1 associated myelopathy or tropical spastic paraparesis (HAM/TSP). Although EV composition is often altered upon viral infection and may participate in inflammation, alterations in EV cargoes have not been addressed in the context of HTLV-1 infection *in vivo*. Indeed, until now, exosome alteration upon HTLV-1 infection has only been characterized for vesicles released *in vitro*, in the supernatant of chronically infected cell lines^7^.

Herein, we aimed to describe the alterations in the protein composition of plasma EVs that occur upon HTLV-1 infection *in vivo*. We found that EVs are more abundant and smaller in the plasma of HTLV-1 infected individuals. These EVs contain proteins derived from mitochondria and lysosomes; they are markers of metabolic and mitochondrial stress.

## Results

### Increased number of small EVs in the plasma of HTLV-1 infected individuals

Extracellular vesicles (EVs) were isolated from the plasma of 5 healthy non-infected individuals (NI), 6 HTLV-1 asymptomatic carriers (HACs), and 5 HAM/TSP patients (Table 1).

**Table 1:** Description of plasma donors.

**NAME**	**AGE**	**STATUS**	**ORIGIN**	**PVL (%)**
NI1	20	NI	African	0
NI2	62	NI	African	0
NI3	67	NI	Caucasian	0
NI4	61	NI	Caucasian	0
NI5	40	NI	Caucasian	0
HAC1	47	HAC	Caribbean	<0.05
HAC2	50	HAC	Caucasian	0.48
HAC3	48	HAC	Caucasian	*n.d*.
HAC4	52	HAC	Caucasian	<0.05
HAC5	30	HAC	Caucasian	0.6
HAC6	68	HAC	Caucasian	*n.d*.
TSP1	68	HAM/TSP	Caucasian	6.7
TSP2	47	HAM/TSP	Caribbean	*n.d*.
TSP3	50	HAM/TSP	Caucasian	*n.d*.
TSP4	66	HAM/TSP	Caucasian	1.2
TSP5	51	HAM/TSP	Caribbean	6.2

The 11 donors were female: 5 were non-infected (NI), 6 were HTLV-1 asymptomatic carriers (HAC), and 5 were HAM/TSP patients. The proviral load (PVL) was determined by quantitative PCR and presented as the number of copies of *tax* per 100 PBMCs. *n.d*. means non-determined (due to absence of DNA).

**Figure 1:**
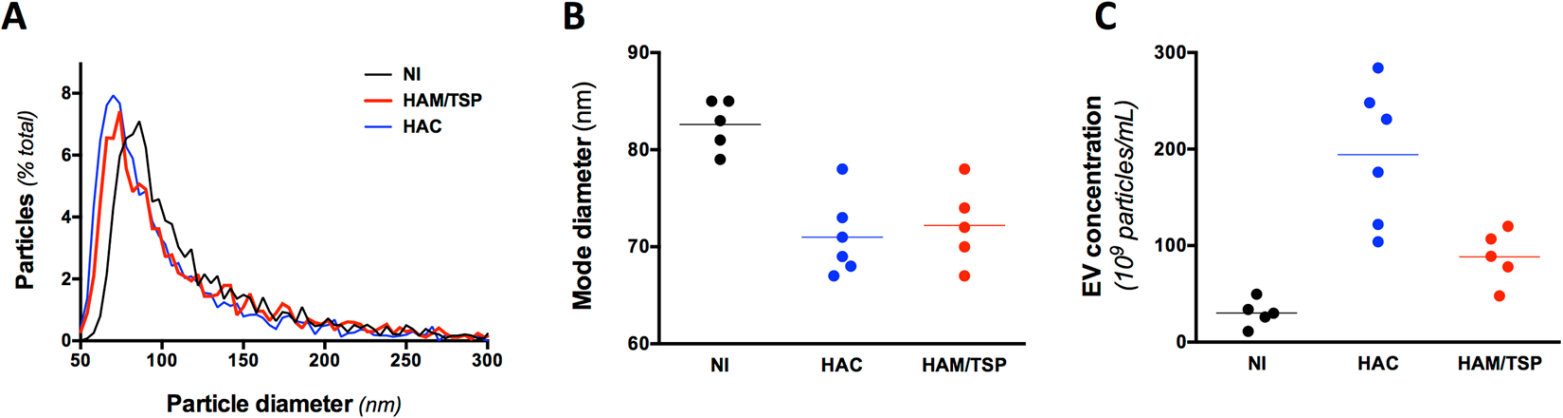
EVs are more abundant and smaller in the plasma of HTLV-1 infected individuals. (**A**) Size distribution of the EV populations. Plasma EVs were isolated on qEV columns and the collected vesicles were studied for size on qNANO. The mean particle size distribution is presented. HD is for healthy donors, HAC for HTLV-1 asymptomatic carriers, and HAM/TSP for patients with HAM/TSP. (**B**) Mode size of plasma EVs. EVs are smaller in the plasma of HTLV-1 infected individuals when compared to NI (p-value = 0,002, Kruskal-Wallis test, Dunn’s post-hoc test). (**C**) EV concentration of the different EV samples. EV concentration was determined with the qNANO device. EV concentration was significantly higher in HAC when compared to NI (p-value < 10^−3^, Kruskal-Wallis test, Dunn’s post-hoc test).

We collected by size exclusion chromatography the plasma EVs with a size comprised between 50 and 150 nm in diameter (Fig. 1A). On average, the size of the EVs isolated from the plasma of HTLV-1 infected individuals was significantly smaller than those of NI donors (Fig. 1A,B).

We also found that plasma from HTLV-1 infected individuals contained significantly more EVs than those from NI donors (Fig. 1C). The plasma EV concentration was the highest for HACs.

### Proteome distinguished between infected and non-infected donors

EV protein content was analyzed by mass spectrometry. For each sample, a large number of peptides (more than 20,000) was detected, which correspond to more than 2,000 identified proteins per sample. A large overlap in exosome protein composition was observed among samples from individuals with similar clinical status (Supplementary Figure 1), demonstrating that inter-individual variations are minor.

Almost a third of all the identified proteins (32,5%) was annotated as belonging to extracellular exosomes (Gene ontology GO:0070062, false discovery rate (FDR) = 10^−88^). Similarly, we detected 92 of the top 100 most frequently identified exosomal proteins, as defined by the Exocarta database. Viral proteins were not detected in any sample.

**Figure 2:**
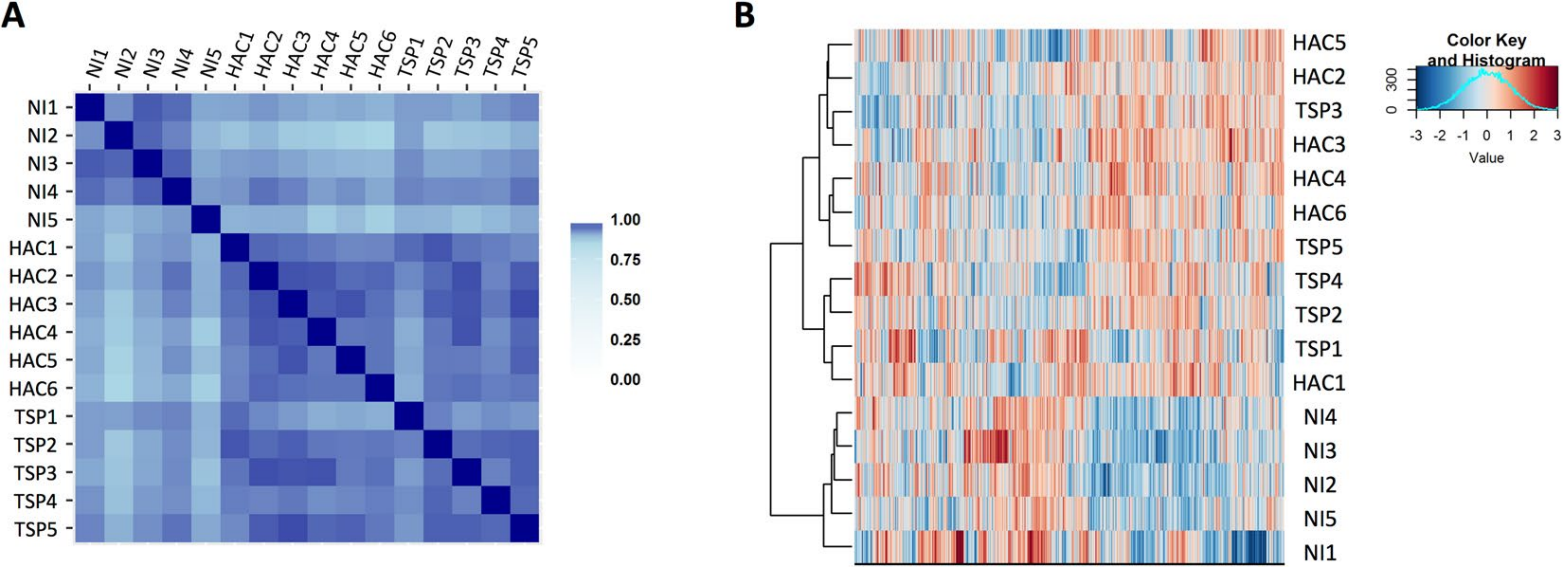
Protein cargo composition distinguishes between HTLV-1 infected donors and non-infected donors. (**A**) Pair-wise correlations between samples. Shades of blue represent correlation values. (**B**) Hierarchical cluster analysis between replicates using Ward’s method with a correlation-based distance.

Next, the LFQ intensities of the EV protein content from the different samples were examined by pairwise correlation (Fig. 2A). Samples from NI individuals displayed high correlation values among them, and presented lower correlation coefficients with samples from HTLV-1 infected individuals. Intriguingly, high correlation values were obtained for the samples derived from HTLV-1 infected individuals, irrespective to the clinical status. Similarly, a hierarchical cluster analysis using Ward’s method and a correlation-based distance was not able to segregate samples from HTLV-1 infected individuals according to the clinical status (HAC *vs* HAM/TSP) (Fig. 2B).

Together, our data analysis of the LFQ intensities suggests that there is a significant protein signature of HTLV-1 infection in the collected EVs. The differences between EV protein composition in HACs and HAM/TSP patients are subtler. Indeed, the strong correlation among samples from HTLV-1 infected individuals indicates that quantified proteins that move specifically in HAC or HAM/TSP represent a small proportion of all the quantified proteins.

### EV protein signature of HTLV-1 infection

**Figure 3:**
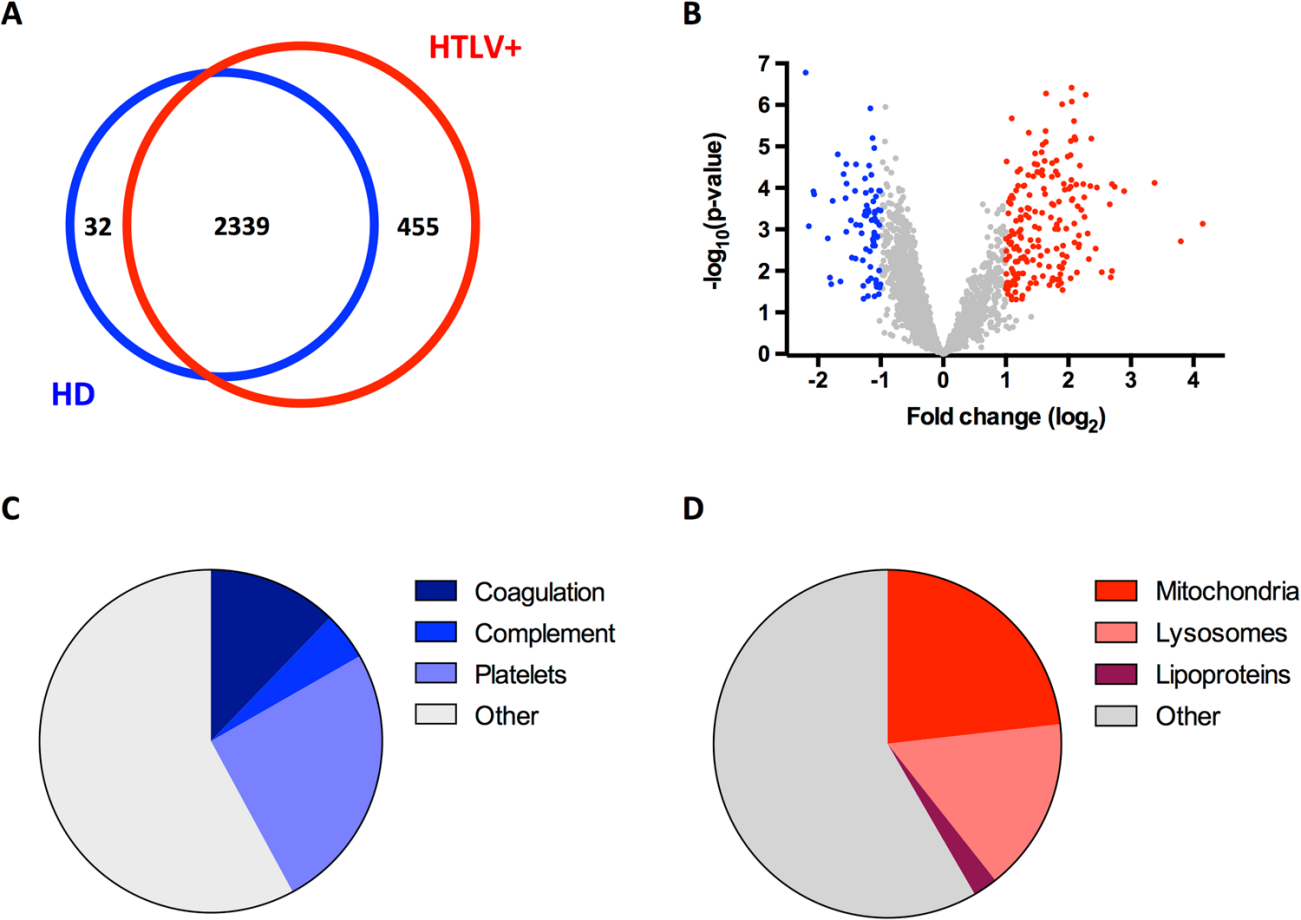
Alterations in protein composition of EVs upon HTLV-1 infection. (**A**) Venn diagram showing the overlap of proteins identified in EVs from NI and HTLV-1 infected individuals. (**B**) Volcano plot representing the quantitative analysis of protein composition of EV from NI and HTLV-1 infected donors. Blue and red dots correspond to proteins more abundant in EVs from HD and HTLV-1 infected individuals, respectively. (**C**) Functional analysis of proteins enriched in EVs from NI. The list of enriched proteins was analyzed with FUNRICH and STRING softwares. The pie chart represents the proportion of proteins associated with coagulation, complement, or the other proteins composing platelet granules. (**D**) Functional analysis of proteins enriched in EVs from HTLV-1 infected donors. The pie chart represents the proportion of proteins present in lipoproteins, lysosomes or mitochondria.

We found that 32 proteins were quantified only in samples from NI, while 455 proteins were quantified only in HTLV patients (Fig. 3A and Supplementary Tables 1, 2). Among the 2339 proteins quantified in both cases (NI and HTLV+), 189 were downregulated and 77 upregulated upon HTLV-1 infection (Fig. 3B and Supplementary Tables 1, 2).

Proteins involved in blood coagulation (25 proteins present in GO:0007596, FDR = 1.5 10^−10^) and complement activation (10 proteins present in GO:0006956, FDR = 3.6 10^−9^) were more abundant in samples from NI (Fig. 3C). These proteins are also components of platelet granules (GO:0031091), suggesting that the decrease we observe was due to a lower proportion of platelet granules among plasma EVs. This hypothesis was supported by the fact that 97 out of 221 proteins that are more abundant in NI had been previously described in platelet-derived vesicles (as presented by Exocarta database). Interestingly, the deficit in platelet-derived proteins is most prevalent in HACs than in HAM/TSP, which mirrors the variations in exosome count (Supplementary Figure 2A).

Among the proteins enriched in samples from HTLV-1 infected individuals, proteins composing plasma lipoproteins (12 proteins in GO:0034358, FDR = 5 10^−9^), lysosomes (85 proteins in GO:0005764, FDR = 1.3 10^−27^) and mitochondria (122 proteins in GO:0005739, FDR = 1.3 10^−27^) were overrepresented (Fig. 3D). Most of the lysosomal and mitochondrial proteins were not quantified in the samples isolated from NI individuals (Supplementary Figure 2B). Moreover, these proteins are often more abundant in samples from HACs than of HAM/TSP patients.

As this trend follows the overall number of EVs, our data suggest that lysosomal and mitochondrial proteins are present together in the small EVs observed upon HTLV-1 infection.

## Discussion

We found that, in HTLV-1 carriers, small EVs are accumulated in the blood stream. These vesicles are enriched in mitochondrial and lysosomal proteins.

It is very likely that these mitochondria- and lysosome-rich EVs are not released from the infected cells, but rather from uninfected cells (or hematopoietic origin or not) in response to viral detection or inflammation. First, HTLV-1 infected cells represent a small portion of the circulating cells (ranging from <0,05 to 10%), and would not be responsible for such an alteration in vesicles count. Moreover, there is no correlation between the amount of altered EVs and the HTLV-1 proviral load (the amount of vesicles in lower in HAM/TSP patients when compared to HTLV-1 carriers). Finally, the composition of these vesicles does not correspond to the composition of EVs secreted by infected cells, as previously determined^7^.

Viral proteins were not detected. The absence of detection of viral proteins was anticipated: HTLV-1 viral expression *in vivo* is low, and, as most of the isolated EVs may have other origins (e.g. platelets, neutrophils, uninfected lymphocytes). However, a meeting abstract suggested that both Tax and HBZ could be detected in exosomes from HAM/TSP patients (but not HACs)^8^. It is unclear whether Tax was not detected in exosomes in our study because of lower proviral loads, differences in disease progression, or technique sensitivity limits.

High levels of EVs are often described upon viral infection. For example, an increase in EV concentration has been reported in HIV viremic individuals^9^. We have also found that EVs present in the plasma of HTLV-1 infected individual are smaller than in NI donors. Changes in the size of an EV population are not uncommon. Smaller EVs have been previously observed in the context of inflammation, cellular stress and tissue damage^10,11^. These smaller vesicles could be either secreted by a particular cell type (that remains to be identified) or correspond to a change in secretion pattern by cells that otherwise secrete larger vesicles. Indeed, a given cell type can release subpopulations of exosomes with different morphology, and properties^12,13^; the relative proportion of these EVs subpopulations may be modified in response to alterations in the microenvironment.

The proportion of platelet-derived vesicles was found diminished on HTLV-1 infection. The decrease in platelet granules is astonishing as HTLV-1 infection has been associated with increased platelet count^14^. Thus, we believe that the apparent diminution in platelet-derived proteins is not due to a deficit in the secretion by platelets, and is rather due to a dilution of these vesicles among virus-induced smaller EVs. This is consistent with the fact that the apparent depletion of platelet-associated proteins is more important for plasma from HAC than of HAM/TSP. Of note, the apparent overexpression of complement and coagulation in HAM/TSP compared to HAC had been previously suggested^8^.

Lipoproteins have been found enriched, when compared to control donors. This is reminiscent of a previous study that found elevated levels of triglyceride and very low-density lipoproteins (VLDL) in HTLV-1 infected women compared with uninfected individuals^15^. Our cohort is mostly composed of female donors. It is unclear whether the fluctuations in lipoproteins occur within exosomes: VLDL overlaps with the size range of EVs or may be adsorbed at the surface of EVs *in vitro*^16^.

The most important protein signature in HTLV-1 infected individuals corresponded to mitochondrial proteins. Such enrichment has not been previously reported, neither on samples from HIV-infected patients^17^ nor in the context of inflammatory diseases (e.g. inflammatory bowel disease, multiple sclerosis)^11,18^. Of note, Dutta *et al*. have not reported such alterations in the context of HTLV infection, likely because they compared the composition of exosomes isolated from the serum of HACs, HAM/TSP and ATL patients, but did not consider the composition of exosomes from NI healthy donors^8^.

Functional mitochondria have been shown to be transferable from cell to cell through vesicles both *in vitro* and *in vivo*. The transfer can occur in stress conditions, and can rescue mitochondrial functions^19^. However, this transfer is usually mediated through large vesicles^19^. In our case, the EVs are much smaller and they also contain lysosomal proteins. Thus, we speculate that these vesicles correspond to extruded mitochondrial in response to inflammation, or cellular stress. *In vitro* it has been reported that, under TNFα-stimulation, Jurkat cells release mitochondria like vesicles, which can be later recognize by innate immune cells and act as a danger signal^20^. This may occur in HTLV-1 infected individuals as they display high plasma levels of inflammatory cytokines^21^. Additionally, oxidative stress may participate in such mitochondrial extrusion: on the one hand, Hela cells extrude fragments of mitochondria under oxidative stress *in vitro*^22^; on the other hand, serum antioxidant capacity is reduced in HTLV-1 infected patients^23^.

However, these mechanisms do not fully explain mitochondrial extrusion in our case. Indeed, while HAM/TSP patients display higher levels of inflammatory cytokines, the concentration in mitochondria- and lysosome-rich EVs is lower in HAM/TSP patients. This suggests that, during HAM/TSP pathogenesis, mitochondrial extrusion is inhibited, or some sort of exhaustion occurs, which eventually counteract inflammatory-induced mitochondria extrusion.

In conclusion, we have demonstrated that EV protein cargo is altered *in vivo* upon HTLV-1 infection, and that they may mirror cellular stress. Such alteration may participate in pathogenesis.

## Methods

### Samples and ethic statement

Plasmas were obtained in the context of a Biomedical Research Program approved by the Committee for the Protection of Persons, Ile-de-France II, Paris (2012-10-04 SC). All individuals gave informed consent. Experiments were performed in accordance with guidelines and regulations.

### Vesicle isolation and characterization

Extracellular vesicles (EVs) were isolated from 1 mL of human plasma by size exclusion chromatography (SEC), using qEV columns (Izon, UK)^24^. Fractions 7 to 9 were pooled, as recommended by the manufacturer. The retrieved vesicles were characterized for concentration and size by Tunable Resistive Pulse Sensing technology, using the qNANO device (IZON)^25^.

### Peptide preparation and Mass Spectrometry

Protein samples were solubilized in urea 8 M, Tris 100 mM pH7.5, then disulfide bonds were reduced with 5 mM dithiothreitol (DTT) for 30 min at 23 °C and alkylated with 20 mM iodoacetamide for 30 min at room temperature in the dark. Subsequently, LysC (Promega) was added for the first digestion step (500 ng) for 3 h at 30 °C. Then the sample was diluted to 1 M urea with 100 mM Tris pH 7.5, and trypsin (Promega) was added to the sample (1 µg for 16 h at 37 °C, then 500 ng for 3 h). Proteolysis was stopped by adding 2% formic acid. Resulting peptides were desalted using Sep-Pak SPE cartridge (Waters) according to manufactures instructions.

LC-MS/MS analysis of digested peptides was performed on an Orbitrap Q Exactive Plus mass spectrometer (Thermo Fisher Scientific, Bremen) coupled to an EASY-nLC 1000 (Thermo Fisher Scientific). Peptides were loaded and separated at 250 nl.min^−1^ on a home-made C18 50 cm capillary column picotip silica emitter tip (75 μm diameter filled with 1.9 μm Reprosil-Pur Basic C18-HD resin, (Dr. Maisch GmbH, Ammerbuch-Entringen, Germany)) equilibrated in solvent A (0.1% FA). Peptides were eluted using a gradient of solvent B (ACN,0.1% FA) from 2% to 5% in 5 min, 5% to 22% in 150 min, 22% to 45% in 60 min (total duration of the chromatographic run was 250 min including high ACN level steps and column regeneration). Mass spectra were acquired in data-dependent acquisition mode with the XCalibur 2.2 software (Thermo Fisher Scientific, Bremen) with automatic switching between MS and MS/MS scans using a top-10 method. MS spectra were acquired at a resolution of 70,000 (at m/z 400) with a target value of 3 × 10^6^ ions. The scan range was limited from 300 to 1,700 m/z. Peptide fragmentation was performed using higher-energy collision dissociation (HCD) with the energy set at 28 NCE. Intensity threshold for ions selection was set at 1 × 10^6^ ions with charge exclusion of z = 1 and z > 7. The MS/MS spectra were acquired at a resolution of 17,500 (at m/z 400). Isolation window was set at 1.6 Th. Dynamic exclusion was employed within 45 s.

Data were searched using MaxQuant (version 1.5.3.8) (with the Andromeda search engine) against a human database (20,202 entries, downloaded from UniProt the 2016.05.25), HTLV-1 viral proteins were searched against a set of 11 entries (including GAG, PRO, POL, REX, TAX, ENV, HBZ, P13, P12, P30 and usHBZ proteins, downloaded from UniProt the 2015.03.12).

The following search parameters were applied: carbamidomethylation of cysteines was set as a fixed modification, oxidation of methionine and protein N-terminal acetylation were set as variable modifications. The mass tolerances in MS and MS/MS were set to 5 ppm and 20 ppm respectively. Maximum peptide charge was set to 7 and 7 amino acids were required as minimum peptide length. A false discovery rate of 1% was set up for both protein and peptide levels. A match between run features was selected between biological replicates with a matched time window set at 1 min and alignment time window set at 20 min. Proteins identified in the reverse and contaminant databases were discarded from the list of identified proteins.

Quantification of the identified proteins was performed using the label free quantification (LFQ) algorithm in MaxQuant^26^.

### Statistical Analysis

The pairwise correlation matrix represents the Pearson correlation coefficients between each pair of samples computed using all complete pairs of LFQ intensity values measured in these samples. The hierarchical cluster analysis has been conducted via multiscale boostrap resampling (1000 bootstrap replications) with the Ward’s method and a correlation-based distance measure thanks to the *pvclust* function of the R package *pvclust*^27^, after log2 transformation of the LFQ intensities, imputation of the missing values with the *imp.norm* function of the R package *norm* and a standardization of the values.

For the statistical analysis of one condition versus another, proteins exhibiting fewer than 3 LFQ values in both conditions were discarded from the list to avoid misidentified proteins. Thus a protein is later referred as quantified in a condition when LFQs have been measured in at least 3 samples of the given condition. After log2 transformation of the leftover proteins, LFQ values were normalized by median centering within conditions (normalizeD function of the R package DAPAR)^28^. Remaining proteins without any LFQ value in one condition have been considered as proteins quantitatively present in a condition and absent in another. They have therefore been set aside and considered as differentially abundant proteins. Next, missing values were imputed using the *imp.norm* function of the R package *norm*. Proteins with a log2 (fold-change) inferior to 1 have been considered as proteins with no significant difference in abundance. Statistical testing of the remaining proteins (having a log2 (fold-change) superior to 1) was conducted using a limma t-test^29^, thanks to the R package *limma*^30^. An adaptive Benjamini-Hochberg procedure was applied on the resulting p-values thanks to the function *adjust.p* of R package *cp4p*^31^ using the robust method of Pounds and Cheng^32^ to estimate the proportion of true null hypotheses among the set of statistical tests. The proteins associated to an adjusted p-value inferior to a FDR level of 1% have been considered as significantly differentially abundant proteins. Finally, the proteins of interest are therefore the proteins that emerge from this statistical analysis supplemented by those being quantitatively absent from one condition and present in another.

### Funtional Enrichement analysis

Enrichment in molecular functions or cellular localization of cargos was analyzed using Funrich^33^ and STRING^34^ databases. The lists of proteins composing exosomes and platelet granules were from Exocarta^35^.

## Electronic supplementary material

Supplementary Information
