# Supplementary material for: Proteomic analysis of plasma extracellular vesicles reveals mitochondrial stress upon HTLV-1 infection
Source: Sci Rep. 2018 Mar 26;8:5170. doi: 10.1038/s41598-018-23505-0 (PMC5980083; doi:10.1038/s41598-018-23505-0)
Supplement: Supplementary file 1 — Supplementary Information [file 41598_2018_23505_MOESM1_ESM.pdf]

SUPPLEMENTAL DATA

Proteomic analysis of plasma extracellular vesicles reveals mitochondrial stress upon HTLV-1 infection

Patricia **Jeannin**, Thibault **Chaze**, Quentin **Giai Gianetto**, Mariette **Matondo**, Olivier **Gout**, Antoine **Gessain**, Philippe V **Afonso**

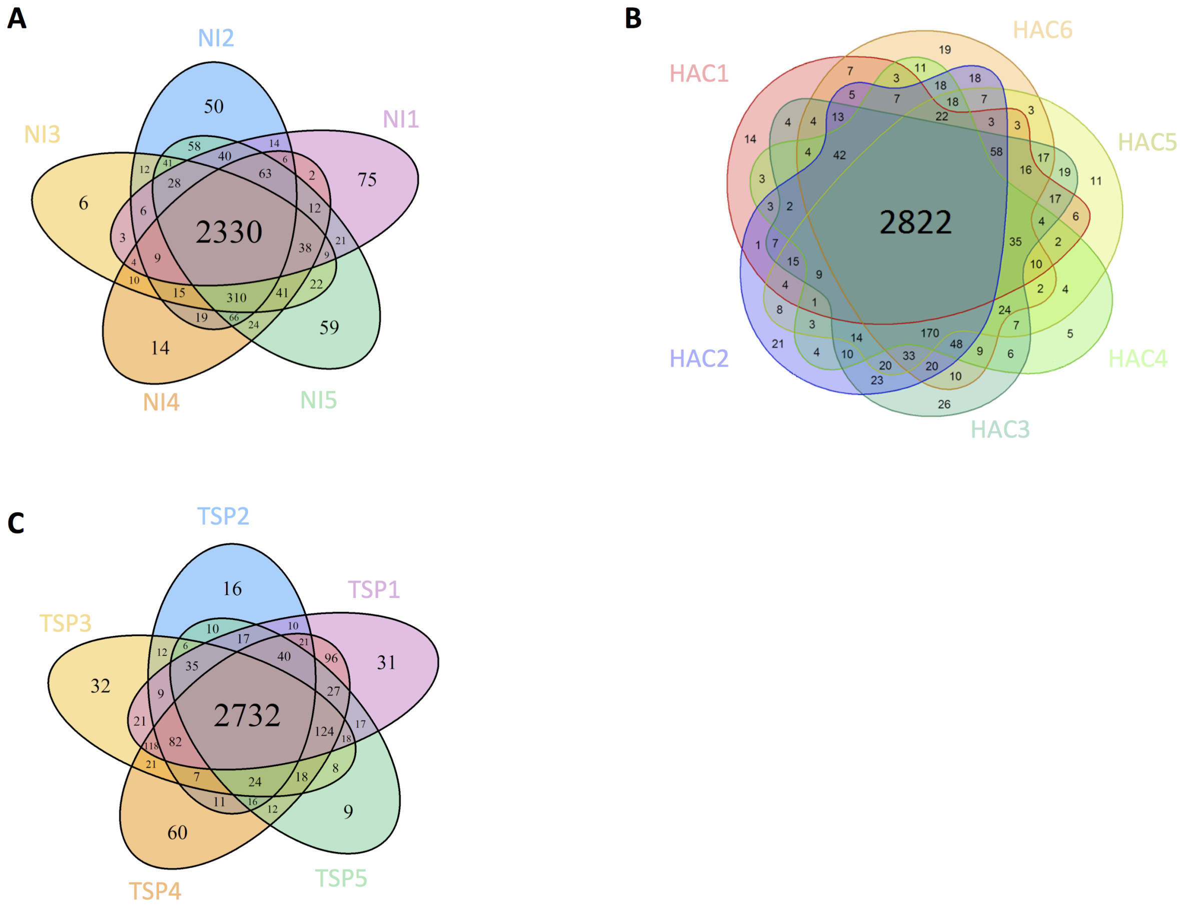

Supplementary figure S1 – Venn diagrams representing the overlap of protiens identified in EVs from NIs (n=5), HACs (n=6) and HAM/TSP patients (n=5).

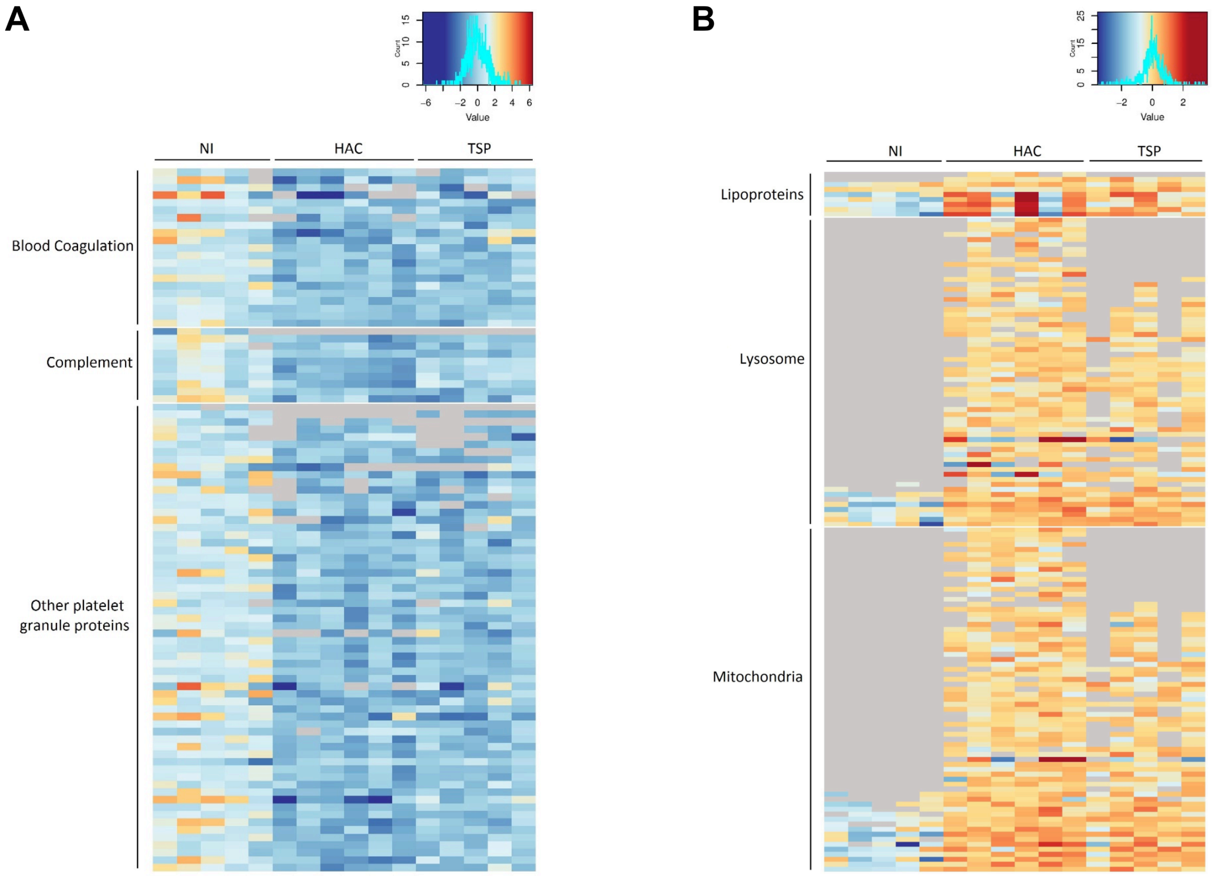

Supplementary Figure S2 – Heatmaps for proteins less (A) and more (B) abundant in plasma from HTLV-positive individuals.

The heatmaps represent the LFQ intensities of proteins, after log2 transformation of the LFQ intensities and centering of the observed values of each protein on their average.

Proteins more abundant in NI samples			
symbol	UniProt	fold increase	Adjusted p-value
PUR9	P31939	2,05	3,7E-06
GLYC	P34896	1,65	3,7E-06
ANGL6	Q8NI99	2,28	3,7E-06
TPIS	P60174	1,90	4,1E-06
S29A1	Q99808	2,06	4,1E-06
6PGD	P52209	1,10	6,7E-06
G6PI	P06744	2,09	6,9E-06
ABLM1	O14639	1,64	1,1E-05
GDIB	P50395	1,37	1,1E-05
AK1A1	P14550	2,37	1,2E-05
MARE1	Q15691	2,11	1,2E-05
PA2G4	Q9UQ80	2,10	1,2E-05
LDHB	P07195	1,64	1,2E-05
TKT	P29401	2,04	1,2E-05
MDHC	P40925	1,59	1,3E-05
LDHA	P00338	1,58	1,8E-05
APOL1	O14791	2,04	1,8E-05
RHG01	Q07960	1,47	1,8E-05
PNPH	P00491	1,99	1,9E-05
C4BPB	P20851	2,18	2,2E-05

MOES	P26038	1,46	2,2E-05
GDIA	P31150	1,01	2,2E-05
TAGL2	P37802	1,62	2,2E-05
GSHB	P48637	1,51	2,2E-05
PA1B2	P68402	1,74	2,2E-05
UT1	Q13336	1,81	2,2E-05
RAP1B	P61224	1,23	2,6E-05
EMAL2	O95834	1,58	2,7E-05
MA1A1	P33908	1,50	2,7E-05
TALDO	P37837	1,80	2,7E-05
PACN2	Q9UNF0	1,19	2,7E-05
A2MG	P01023	1,53	2,7E-05
PROF1	P07737	1,83	2,9E-05
RALB	P11234	1,59	2,9E-05
NSF1C	Q9UNZ2	1,36	2,9E-05
LSM1	O15116	1,44	2,9E-05
TYPH	P19971	1,74	3,0E-05
PRDX2	P32119	2,05	3,4E-05
TFR1	P02786	2,70	3,8E-05
CO6A1	P12109	2,24	3,8E-05
LBP	P18428	3,38	3,8E-05
CD81	P60033	1,93	3,8E-05
GOLI4	O00461	2,13	3,9E-05

LV301	P01714	2,35	3,9E-05
CNPY3	Q9BT09	1,30	3,9E-05
COL11	Q9BWP8	1,28	3,9E-05
PARVG	Q9HBI0	1,20	3,9E-05
HV311	P01772	2,04	3,9E-05
LG3BP	Q08380	2,74	3,9E-05
KV110	P01602	2,46	4,1E-05
TPD54	O43399	1,61	4,2E-05
HPTR	P00739	2,01	4,2E-05
HV319	P01780	1,94	4,3E-05
CETP	P11597	2,89	4,3E-05
NIT2	Q9NQR4	1,17	4,3E-05
HV206	P01824	1,63	4,9E-05
PGAM1	P18669	1,38	5,0E-05
ISOC1	Q96CN7	1,09	5,2E-05
MFGM	Q08431	2,25	5,5E-05
FIBA	P02671	1,07	5,5E-05
CAB39	Q9Y376	1,11	5,5E-05
FLNC	Q14315	2,07	5,7E-05
HBB	P68871	2,06	6,0E-05
RELN	P78509	1,85	6,3E-05
VINC	P18206	1,05	6,3E-05
GDIR1	P52565	1,08	6,9E-05

GDIR2	P52566	1,74	6,9E-05
C4BPA	P04003	2,66	7,0E-05
MASP1	P48740	1,76	7,2E-05
HV302	P01763	2,15	7,5E-05
GMPR1	P36959	1,35	8,2E-05
CD34	P28906	1,37	8,4E-05
CO9	P02748	1,24	8,7E-05
TENA	P24821	2,21	8,7E-05
SYUA	P37840	1,47	9,2E-05
SODC	P00441	1,58	9,6E-05
F13A	P00488	1,04	9,8E-05
PROS	P07225	1,32	1,1E-04
KELL	P23276	2,26	1,1E-04
RHCE	P18577	1,37	1,1E-04
SORCN	P30626	1,24	1,1E-04
S10AC	P80511	1,72	1,1E-04
UXS1	Q8NBZ7	1,24	1,3E-04
VWF	P04275	1,86	1,3E-04
PICAL	Q13492	1,16	1,3E-04
LV101	P01699	2,10	1,5E-04
FA11	P03951	4,15	1,5E-04
GLPA	P02724	1,81	1,5E-04
C1R	P00736	1,46	1,7E-04

ANXA7	P20073	1,53	1,8E-04
CO6A3	P12111	1,97	1,9E-04
FINC	P02751	1,79	1,9E-04
HBA	P69905	1,83	1,9E-04
PTPA	Q15257	1,24	1,9E-04
MARE2	Q15555	1,09	2,1E-04
ALG5	Q9Y673	1,21	2,1E-04
PRG4	Q92954	2,31	2,3E-04
BLVRB	P30043	1,13	2,3E-04
CD5L	O43866	2,17	2,5E-04
RET4	P02753	1,06	2,6E-04
PAIP1	Q9H074	1,23	2,6E-04
RSSA	P08865	1,25	2,7E-04
FIBG	P02679	1,00	2,8E-04
IGKC	P01834	1,52	3,0E-04
TYB4	P62328	1,39	3,0E-04
FCGBP	Q9Y6R7	3,80	3,2E-04
CAH1	P00915	2,10	3,3E-04
CLIC4	Q9Y696	1,05	3,3E-04
MARCS	P29966	1,09	3,3E-04
SHOC2	Q9UQ13	1,08	3,6E-04
IV4F8	A6NJ16	2,17	3,7E-04
KV402	P01625	1,92	3,9E-04

SH3L1	O75368	1,44	4,0E-04
PP1R7	Q15435	1,17	4,0E-04
LAC2	P0CG05	1,52	4,0E-04
PIGR	P01833	2,17	4,1E-04
LV104	P01702	2,44	4,3E-04
IGHM	P01871	1,81	4,3E-04
AT2B4	P23634	1,13	4,3E-04
TIMP1	P01033	1,21	4,6E-04
HV320	P01781	1,66	4,8E-04
TFIP8	O95379	1,01	4,8E-04
FA12	P00748	1,35	5,0E-04
COTL1	Q14019	1,90	5,8E-04
ANXA5	P08758	1,56	6,2E-04
LYSC	P61626	1,06	6,3E-04
IGJ	P01591	1,97	6,4E-04
SAMP	P02743	1,29	6,5E-04
CAH2	P00918	1,24	6,8E-04
ATS13	Q76LX8	2,33	7,1E-04
C1QC	P02747	1,32	7,2E-04
PSB7	Q99436	1,01	7,2E-04
PP14A	Q96A00	1,00	7,3E-04
RAB4A	P20338	1,43	7,3E-04
ENOG	P09104	1,19	7,9E-04

APT	P07741	1,34	7,9E-04
TEBP	Q15185	1,16	7,9E-04
LV102	P01700	1,93	8,3E-04
KV206	P06310	1,54	9,4E-04
SRCRL	A1L4H1	1,86	1,0E-03
KV305	P01623	1,69	1,0E-03
HV303	P01764	1,90	1,1E-03
H90B2	Q58FF8	1,10	1,1E-03
HV304	P01765	2,70	1,3E-03
CXCL7	P02775	1,11	1,3E-03
HV316	P01777	2,53	1,3E-03
FA8	P00451	2,14	1,4E-03
41	P11171	1,12	1,4E-03
LV302	P80748	1,26	1,4E-03
POTEE	Q6S8J3	1,28	1,4E-03
RD23B	P54727	1,20	1,4E-03
CY24B	P04839	1,01	1,4E-03
LV001	P04211	1,87	1,4E-03
C1QA	P02745	1,14	1,5E-03
F13B	P05160	1,59	1,6E-03
OIT3	Q8WWZ8	2,68	1,7E-03
IGLL5	B9A064	1,45	1,7E-03
BLMH	Q13867	1,75	1,7E-03

RARR2	Q99969	1,19	1,7E-03
LV403	P01717	2,04	1,7E-03
C1QB	P02746	1,16	1,8E-03
KV313	P18136	1,85	1,8E-03
HEG1	Q9ULI3	1,82	1,8E-03
HBD	P02042	1,74	1,9E-03
KV309	P04433	1,13	1,9E-03
LV106	P04208	1,05	2,1E-03
GTR1	P11166	1,02	2,1E-03
HV306	P01767	1,89	2,2E-03
MUCB	P04220	1,48	2,2E-03
HV209	P06331	1,40	2,2E-03
1A03	P04439	1,15	2,3E-03
LV405	P06889	1,70	2,3E-03
HV103	P23083	1,08	2,4E-03
KV303	P01621	1,84	2,4E-03
EPB42	P16452	1,01	2,4E-03
ANXA2	P07355	1,03	2,6E-03
ADDB	P35612	1,36	2,7E-03
ITIH3	Q06033	1,00	2,8E-03
SYYC	P54577	1,03	2,9E-03
C1QT3	Q9BXJ4	1,91	2,9E-03
LV205	P01708	1,16	3,3E-03

IGHA2	P01877	1,03	3,6E-03
ANXA6	P08133	1,27	3,9E-03
ERMAP	Q96PL5	1,09	4,1E-03
KV118	P01610	1,26	4,6E-03
GLPC	P04921	1,09	4,8E-03
CD14	P08571	1,16	4,8E-03
ATIF1	Q9UII2	1,52	5,9E-03
KV307	P04206	1,05	7,1E-03

**Proteins detected exclusively in NI samples**	
BT3A1	O00481
PODXL	O00592
TXND9	O14530
SEM7A	O75326
RASK	P01116
ICAM1	P05362
CHLE	P06276
MBL2	P11226
MIF	P14174
AT2B1	P20020
ITAL	P20701
TGM2	P21980
DOPD	P30046

HINT1	P49773
BCAM	P50895
CRIP2	P52943
DCD	P81605
RHD	Q02161
2A5A	Q15172
NHRF2	Q15599
AAAT	Q15758
PPR18	Q6NYC8
SRCAP	Q6ZRS2
HMCN2	Q8NDA2
TITIN	Q8WZ42
ARL8A	Q96BM9
GMPR2	Q9P2T1
NUDT5	Q9UKK9
UBQL1	Q9UMX0
CHIP	Q9UNE7
MEMO1	Q9Y316
NPTN	Q9Y639

Supplementary table S1 – List of proteins that were more abundant in samples from NI than in samples from HTLV-1 infected individuals.

For each protein, the symbol, UniProt accession number, log2(foldchange)(i.e. Log2(mean LFQ HTLV/mean LFQ NI) and the adjusted p-value are presented.

Proteins more abundant in HTLV+ samples			
**symbol**	**UniProt**	**fold increase**	**Adjusted p-value**
AL4A1	P30038	-2,20	3,7E-06
NDUS8	O00217	-1,17	4,4E-06
SSFA2	P28290	-1,13	1,2E-05
RNF31	Q96EP0	-1,10	1,5E-05
VTDB	P02774	-1,68	1,8E-05
DEN2C	Q68D51	-1,18	2,2E-05
K1211	Q6ZU35	-1,39	2,2E-05
PP6R1	Q9UPN7	-1,55	2,2E-05
UBA6	A0AVT1	-1,59	2,9E-05
NOMO1	Q15155	-1,15	2,9E-05
ODB2	P11182	-1,25	3,2E-05
LRMP	Q12912	-1,55	3,8E-05
HYES	P34913	-1,15	4,3E-05
S35E1	Q96K37	-1,02	4,3E-05
SLK	Q9H2G2	-1,01	4,3E-05
EMRE	Q9H4I9	-2,07	4,3E-05
ARHGC	Q9NZN5	-1,41	4,3E-05
CD63	P08962	-1,24	4,6E-05
MYCB2	O75592	-2,06	4,9E-05
F16B1	Q5W0V3	-1,07	5,3E-05

FAKD5	Q7L8L6	-1,56	5,5E-05
S39A7	Q92504	-1,77	6,2E-05
S2535	Q3KQZ1	-1,12	6,3E-05
BIG2	Q9Y6D5	-1,22	7,3E-05
CBL	P22681	-1,04	8,7E-05
LPPRC	P42704	-1,20	8,7E-05
NDUB4	O95168	-1,00	9,0E-05
P5CS	P54886	-1,24	9,2E-05
COX5A	P20674	-1,09	9,2E-05
SCMC1	Q6NUK1	-1,19	9,3E-05
ECHP	Q08426	-1,10	9,8E-05
EEA1	Q15075	-1,26	1,1E-04
RHG10	A1A4S6	-1,23	1,1E-04
ACO13	Q9NPJ3	-1,09	1,2E-04
ACAD9	Q9H845	-1,15	1,3E-04
VPS25	Q9BRG1	-1,48	1,3E-04
IKKA	O15111	-1,08	1,3E-04
CACO1	Q9P1Z2	-1,06	1,4E-04
VIME	P08670	-1,39	1,5E-04
ACAP2	Q15057	-1,02	1,6E-04
IKKB	O14920	-1,32	1,6E-04
ACADS	P16219	-2,15	1,6E-04
UACA	Q9BZF9	-1,55	2,1E-04

MAP1S	Q66K74	-1,10	2,2E-04
NAGAB	P17050	-1,30	2,2E-04
PFD1	O60925	-1,10	2,5E-04
ACSL3	O95573	-1,05	2,6E-04
IVD	P26440	-1,06	2,6E-04
GPX3	P22352	-1,84	2,8E-04
VPS39	Q96JC1	-1,12	2,9E-04
S23IP	Q9Y6Y8	-1,11	3,2E-04
PDK1	Q15118	-1,12	3,9E-04
UBP24	Q9UPU5	-1,09	3,9E-04
COX7C	P15954	-1,24	4,4E-04
LIPL	P06858	-1,17	4,9E-04
APOC1	P02654	-1,46	6,6E-04
CLD3	O15551	-1,40	6,9E-04
PNCB	Q6XQN6	-1,29	7,4E-04
TSPOA	P30536	-1,17	1,0E-03
ZN363	Q96PM5	-1,02	1,2E-03
APOC4	P55056	-1,81	1,7E-03
ANGI	P03950	-1,16	1,7E-03
TTC27	Q6P3X3	-1,07	1,9E-03
APOE	P02649	-1,20	2,0E-03
SAA4	P35542	-1,64	2,0E-03
ATP8	P03928	-1,07	2,2E-03

APOB	P04114	-1,79	2,3E-03
ANR28	O15084	-1,00	2,3E-03
TENS1	Q9HBL0	-1,28	2,4E-03
S35B4	Q969S0	-1,04	2,4E-03
QOR	Q08257	-1,06	2,5E-03
HLAE	P13747	-1,02	2,6E-03
JAK3	P52333	-1,03	3,7E-03
APOD	P05090	-1,20	4,0E-03
CAMP	P49913	-1,10	4,1E-03
PERM	P05164	-1,27	4,6E-03
DEF1	P59665	-1,21	9,8E-03
**Proteins detected exclusively in HTLV+ samples**			
SYTC2	A2RTX5		
GSAP	A4D1B5		
XIRP2	A4UGR9		
BTBDB	A6QL63		
GPHRB	P0CG08		
WASH3	C4AMC7		
CG073	E0CX11		
ODPX	O00330		
PDE2A	O00408		
NDKM	O00746		
DHSD	O14521		

COX7R	O14548
ACPM	O14561
CDIPT	O14735
TSN4	O14817
OPLA	O14841
BCKD	O14874
UB2L6	O14933
DSCR3	O14972
S27A2	O14975
ADEC1	O15204
BCAT2	O15382
PAPS1	O43252
ZW10	O43264
EMC8	O43402
FIBP	O43427
HTRA2	O43464
MAAI	O43708
AAKB2	O43741
NRDC	O43847
TIM8A	O60220
GMDS	O60547
TSN2	O60636
PP6R2	O75170

COQ9	O75208
NDUB1	O75438
CRTAP	O75718
CMC1	O75746
RENR	O75787
AP1G2	O75843
B3GA3	O94766
SC24D	O94855
PROSC	O94903
GLCE	O94923
NDUB6	O95139
ZFPL1	O95159
NDUA3	O95167
VAMP5	O95183
GOSR1	O95249
NDUC2	O95298
ATG7	O95352
VNN1	O95497
NFAC1	O95644
PPR3D	O95685
RAB3D	O95716
TM50A	O95807
BAG2	O95816

M4K4	O95819
DCMC	O95822
CF047	O95873
RECK	O95980
CYB5	P00167
PDGFB	P01127
NU4M	P03905
NU5M	P03915
LCAT	P04180
KV123	P04431
KV401	P06312
HEXA	P06865
HYEP	P07099
FES	P07332
CATB	P07858
CATG	P08311
VILI	P09327
RO60	P10155
COX8A	P10176
H12	P16403
TFPI1	P10646
PPAL	P11117
SYHC	P12081

IDE	P14735
B4GT1	P15291
PHKG2	P15735
PP2BB	P16298
NCK1	P16333
NCPR	P16435
BPI	P17213
NDUB7	P17568
ML12A	P19105
GGT1	P19440
E2AK2	P19525
KCNA3	P22001
MUTA	P22033
KPCL	P24723
ARBK1	P25098
MGAT1	P26572
VATL	P27449
CD38	P28907
SHC1	P29353
SC6A6	P31641
AKT1	P31749
HNRH1	P31943
ABCD1	P33897

CTNB1	P35222
CDN1A	P38936
MPV17	P39210
IL6RB	P40189
NAA10	P41227
MTHR	P42898
NEDD4	P46934
TPC10	P48553
PRC2A	P48634
CAMLG	P49069
FMO5	P49326
EI2BB	P49770
TSC2	P49815
AP4A	P50583
RL14	P50914
FXR1	P51114
RB27A	P51159
CLCN7	P51798
NUBP1	P53384
ANAG	P54802
S12A2	P55011
MFAP4	P55083
STX17	P56962

SYJ2B	P57105
GSDMD	P57764
CXCR4	P61073
UFM1	P61960
DCAF7	P61962
RS16	P62249
RS18	P62269
RS11	P62280
RB11A	P62491
RS4X	P62701
VAMP2	P63027
BL1S1	P78537
GABT	P80404
COG7	P83436
PGBM	P98160
ILRL1	Q01638
BDH	Q02338
MP2K1	Q02750
PLOD1	Q02809
ATP7A	Q04656
VMAT2	Q05940
KDSR	Q06136
GFPT1	Q06210

GOGA3	Q08378
APOBR	Q0VD83
GALT1	Q10472
STRUM	Q12768
TRAF2	Q12933
SEC20	Q12981
NFX1	Q12986
PAFA	Q13093
PDZ1I	Q13113
LIPA1	Q13136
NDK3	Q13232
2A5G	Q13362
TMED1	Q13445
RIPK1	Q13546
KCC2G	Q13555
SNX1	Q13596
CUL1	Q13616
CD166	Q13740
AUHM	Q13825
UBE4A	Q14139
BECN1	Q14457
ITPR3	Q14573
COG2	Q14746

RB39A	Q14964
PDK3	Q15120
EBP	Q15125
PMVK	Q15126
NONO	Q15233
TAF1A	Q15573
TRADD	Q15628
TRAM1	Q15629
TRIP6	Q15654
TBCE	Q15813
ETFD	Q16134
CCDC6	Q16204
LAMA4	Q16363
KGUA	Q16774
1C16	Q29960
CL16A	Q2KHT3
K1109	Q2LD37
IAH1	Q2TAA2
GRDN	Q3V6T2
GTDC1	Q4AE62
NAKD2	Q4G0N4
TM41B	Q5BJD5
HERC4	Q5GLZ8

MIA3	Q5JRA6
COX20	Q5RI15
PPM1L	Q5SGD2
ODR4	Q5SWX8
STRP1	Q5VSL9
AB17B	Q5VST6
FOCAD	Q5VW36
SPRY7	Q5W111
PTRD1	Q6GMV3
SIGIR	Q6IA17
NADE	Q6IA69
PDE12	Q6L8Q7
RINT1	Q6NUQ1
ARMC6	Q6NXE6
TAPT1	Q6NXT6
CH082	Q6P1X6
FAHD1	Q6P587
LARP1	Q6PKG0
APOA5	Q6Q788
BL1S3	Q6QNY0
RICTR	Q6R327
ATRAP	Q6RW13
AFTIN	Q6ULP2

LCLT1	Q6UWP7
PERQ2	Q6Y7W6
BNC2	Q6ZN30
RINL	Q6ZS11
TMPPE	Q6ZT21
CO052	Q6ZUT6
UBP31	Q70CQ4
MOB2	Q70IA6
MOB3C	Q70IA8
MRRP1	Q7L0Y3
ASGL1	Q7L266
GET4	Q7L5D6
HS2ST	Q7LGA3
MICA3	Q7RTP6
TPC11	Q7Z392
CC186	Q7Z3E2
CP062	Q7Z3J2
BRAP	Q7Z569
CCD91	Q7Z6B0
GP180	Q86V85
VPS36	Q86VN1
PACS2	Q86VP3
SESD1	Q86VW0

RLGPB	Q86X10
COMD2	Q86X83
NDUAB	Q86Y39
DOPP1	Q86YN1
C2CD5	Q86YS7
NNRD	Q8IW45
MFN1	Q8IWA4
TEX2	Q8IWB9
Z3H7A	Q8IWR0
MIRO1	Q8IXI2
K2013	Q8IYS2
AN13A	Q8IZ07
ELMD2	Q8IZ81
SPG20	Q8N0X7
ABD12	Q8N2K0
ZADH2	Q8N4Q0
CBR4	Q8N4T8
AFAP1	Q8N556
OXR1	Q8N573
FA89B	Q8N5H3
F101B	Q8N5W9
COMD1	Q8N668
ARFG1	Q8N6T3

GT251	Q8NBJ5
UBAC2	Q8NBM4
RDH13	Q8NBN7
FA63B	Q8NBR6
ATAD1	Q8NBU5
MTMRE	Q8NCE2
PDPR	Q8NCN5
RN214	Q8ND24
MROH1	Q8NDA8
PK3C3	Q8NEB9
FAD1	Q8NFF5
ZNRF2	Q8NHG8
NCOA7	Q8NI08
GDAP1	Q8TB36
S35B2	Q8TB61
S2540	Q8TBP6
KISHA	Q8TBQ9
PI42C	Q8TBX8
TM163	Q8TC26
C99L2	Q8TCZ2
BL1S5	Q8TDH9
RHBL4	Q8TEB9
AF1L1	Q8TED9

SNX29	Q8TEQ0
WHAMM	Q8TF30
COG1	Q8WTW3
SMAP2	Q8WU79
TB22A	Q8WUA7
TSK	Q8WUA8
ZFN2B	Q8WV99
ATX2L	Q8WWM7
FHR4	Q92496
FAM3C	Q92520
NICA	Q92542
TM9S4	Q92544
HPS1	Q92902
GCDH	Q92947
MYDGF	Q969H8
RRF2M	Q969S9
RFT1	Q96AA3
OTUL	Q96BN8
TBC20	Q96BZ9
F136A	Q96C01
ACSF2	Q96CM8
KCD12	Q96CX2
REPS1	Q96D71

LEG12	Q96DT0
YIPF6	Q96EC8
MMAB	Q96EY8
SIPA1	Q96FS4
FAH2A	Q96GK7
CQ059	Q96GS4
FXL20	Q96IG2
ITCH	Q96J02
VPS50	Q96JG6
VCIP1	Q96JH7
RN170	Q96K19
CFA53	Q96M91
COG8	Q96MW5
MBOA7	Q96N66
RILP	Q96NA2
SFXN2	Q96NB2
ANR27	Q96NW4
LRRC7	Q96NW7
TPPC9	Q96Q05
MCCA	Q96RQ3
CP013	Q96S19
VAT1	Q99536
T22D3	Q99576

TSNAX	Q99598
THIOM	Q99757
ATP5S	Q99766
ARP5L	Q9BPX5
SIN1	Q9BPZ7
CPPED	Q9BRF8
TRI56	Q9BRZ2
TACO1	Q9BSH4
GCP2	Q9BSJ2
MALD1	Q9BSK0
LIMD2	Q9BT23
LMF2	Q9BU23
MIC26	Q9BUR5
RN126	Q9BV68
MECR	Q9BV79
LST8	Q9BVC4
PTSS2	Q9BVG9
FUND2	Q9BWH2
TPSNR	Q9BX59
B2L13	Q9BXK5
RM04	Q9BYD3
DPY30	Q9C005
TRIM4	Q9C037

UNK	Q9C0B0
XPO4	Q9C0E2
CEP44	Q9C0F1
COMD5	Q9GZQ3
T126A	Q9H061
COMD4	Q9H0A8
SHRPN	Q9H0F6
RAB6C	Q9H0N0
TM222	Q9H0R3
TARA	Q9H2D6
CJ011	Q9H2I8
PTN23	Q9H3S7
RABE2	Q9H5N1
HN1L	Q9H910
COG4	Q9H9E3
NHEJ1	Q9H9Q4
GRPE1	Q9HAV7
RRAGC	Q9HB90
ZFYV1	Q9HBF4
PLRKT	Q9HBL7
GLOD4	Q9HC38
MRS2	Q9HD23
GOPC	Q9HD26

SYSM	Q9NP81
RT30	Q9NP92
EMC7	Q9NPA0
OSGEP	Q9NPF4
INO1	Q9NPH2
RIC8A	Q9NPQ8
GP108	Q9NPR9
HINT3	Q9NQE9
HPS4	Q9NQG7
PRDC1	Q9NRG1
ABCBA	Q9NRK6
ADPPT	Q9NRN7
OSTC	Q9NRP0
ABC3C	Q9NRW3
ACER3	Q9NUN7
LIN7C	Q9NUP9
PANK4	Q9NVE7
NECP2	Q9NVZ3
BSDC1	Q9NW68
OXSM	Q9NWU1
ABI2	Q9NYB9
FKB11	Q9NYL4
TECR	Q9NZ01

GGA3	Q9NZ52
GDE1	Q9NZC3
PD1L1	Q9NZQ7
CHMP5	Q9NZZ3
EMC3	Q9P0I2
MARH2	Q9P0N8
TOM7	Q9P0U1
VPS54	Q9P1Q0
JCAD	Q9P266
SPAST	Q9UBP0
SE1L1	Q9UBV2
PEF1	Q9UBV8
FBLN5	Q9UBX5
CS025	Q9UFG5
FETUB	Q9UGM5
SEC63	Q9UGP8
CHRD1	Q9UHD1
PCYOX	Q9UHG3
EI2BD	Q9UI10
ACAD8	Q9UKU7
MYO5B	Q9ULV0
SYNRG	Q9UMZ2
FAF1	Q9UNN5

PPIE	Q9UNP9
COG5	Q9UP83
CP131	Q9UPN4
HDAC5	Q9UQL6
BCS1	Q9Y276
ITM2B	Q9Y287
AKAP2	Q9Y2D5
DJC16	Q9Y2G8
CHSP1	Q9Y2V2
COG6	Q9Y2V7
SNX24	Q9Y343
ORN	Q9Y3B8
CCD53	Q9Y3C0
GOT1B	Q9Y3E0
CHMP3	Q9Y3E7
TMED3	Q9Y3Q3
HBS1L	Q9Y450
DMXL1	Q9Y485
FA65B	Q9Y4F9
ATG4B	Q9Y4P1
PRC2C	Q9Y520
PPME1	Q9Y570
AP4S1	Q9Y587

TIM13	Q9Y5L4
IR3IP	Q9Y5U9
NFS1	Q9Y697
COMDA	Q9Y6G5
CEPT1	Q9Y6K0
STON1	Q9Y6Q2
CAN7	Q9Y6W3

Supplementary table S2 – List of proteins that were statistically more abundant in samples from HTLV+ samples that in samples from NI donors.

For each protein, the symbol, UniProt accession number, log2(foldchange)(i.e. Log2(mean LFQ HTLV/mean LFQ NI) and the adjusted p-value are presented.
